# Health Benefits of Physical Activity: A Strengths-Based Approach

**DOI:** 10.3390/jcm8122044

**Published:** 2019-11-21

**Authors:** Darren E. R. Warburton, Shannon S. D. Bredin

**Affiliations:** 1 Cardiovascular Physiology and Rehabilitation Laboratory, Indigenous Studies in Kinesiology, University of British Columbia, Vancouver, BC V6T1Z4, Canada; 2 Physical Activity Promotion and Chronic Disease Prevention Unit, University of British Columbia, Vancouver, BC V6T1Z4, Canada; shannon.bredin@ubc.ca; 3 Laboratory for Knowledge Mobilization, University of British Columbia, Vancouver, BC V6T1Z4, Canada

**Keywords:** strengths-based, physical activity, exercise, cardiac rehabilitation, wholistic, wellness, health, wellbeing, health promotion, hope

## Abstract

Our special series on Cardiac Rehabilitation outlined the importance of routine physical activity and/or exercise participation in the primary and secondary prevention of cardiovascular disease and many other chronic medical conditions. The evidence is overwhelming, demonstrating that nearly everyone can benefit from becoming more physically active. This messaging has been widely disseminated at regional, national, and international levels. Often, this messaging highlights a physical inactivity crisis and the health perils of not engaging in sufficient amounts of physical activity. This deficits-based messaging often includes generic threshold-based recommendations stating that health benefits can only be accrued with specific volumes or intensities of physical activity. In this Editorial, we argue that the current generic and deficits-based messaging misses a great opportunity to focus on the positive and to facilitate hope and real change at the individual, community, and population levels. We advocate a strengths-based approach to health and wellness promotion that focuses on the innate strengths of individuals, families, and communities to enable self-empowerment and self-determination related to health and wellness. By taking a strengths-based approach, we can build hope, promoting the positive aspects of routine physical activity and exercise participation and providing a greater opportunity to enhance health and wellbeing for everyone.

## 1. Introduction

The health benefits of routine physical activity and exercise participation are irrefutable [1,2,3,4,5,6]. Virtually everyone can benefit from becoming more physically active [2]. Various national and international organizations have provided physical activity recommendations across the lifespan [7,8,9,10,11,12], including recommendations for persons living with chronic medical conditions [13,14,15,16]. Concurrent with these guidelines and recommendations are often statements regarding the health perils associated with being physically inactive. Often, the negative health effects of physical inactivity are emphasized strongly to support the need for routine physical activity. Moreover, generic recommendations are often provided that fail to take into account the unique attributes of each individual. In this Editorial, we argue how this deficits-based approach to physical activity promotion may actually lead to unintended and undesirable results with respect to health and wellness at the individual, family, community, and societal levels. We advocate for a strengths-based approach to health and wellness promotion that builds upon the innate strengths and aspirations of individuals, families, and communities.

## 2. Brief Summary of the Evidence

Numerous systematic reviews of the literature have supported the importance of routine physical activity and/or exercise participation for the primary and secondary prevention of diseases of the cardiovascular system (in particular) and many other chronic medical conditions [2]. Regular physical activity and/or exercise participation is thought to be of benefit for more than 25 chronic medical conditions [1,2,3,4,5,6,17,18]. There are well-established dose–response relationships between physical activity and health with consistent 20–30% risk reductions for premature mortality and several chronic medical conditions [4,18,19,20]. Larger risk reductions are generally observed when objective markers of aerobic fitness are considered [2,4,20,21]. In a recent systematic review of systematic reviews of the literature [2], we revealed compelling data (from millions of participants) that routine physical activity was associated in dose-dependent fashion with a reduced risk for diverse health outcomes (such as cardiovascular disease, all-cause mortality, all-cancer mortality, type 2 diabetes, hypertension, breast cancer, colon cancer, gestational diabetes, gallstone disease, ischemic heart disease, ischemic stroke, and self-reported health status). In the vast majority of the studies, there was a non-linear relationship, with the greatest relative health benefits seen with relatively minor changes in physical activity participation in inactive individuals. This systematic review also revealed that no minimal threshold existed for these health benefits. Importantly, we demonstrated that “simply moving more led to significant health benefits” [2]. The level of activity required for health benefits was far below many current national and international physical activity recommendations. These findings have been supported by various recent epidemiological studies [22,23,24]. Importantly, these health benefits appear to cover a wide range of medical conditions including mental health and wellness. For instance, Harvey and colleagues [24] recently revealed that regular leisure-time exercise of any intensity was associated with a reduced risk for the development of depression in apparently healthy adults (over a 11-year period). After adjusting for a series of confounders, the authors estimated that approximately 12% of future cases of depression could be prevented if participants engaged in at least 1 h of physical activity/exercise each week. A recent systematic review and meta-analysis of 14 studies (from six prospective cohorts) revealed that running participation was associated with a 27, 30, and 23%, respectively, reduced risk of all-cause-, cardiovascular-, and cancer-related mortality. Importantly, very small doses of running (i.e., ≤1 time a week, <50 min a week, <6 mph, and <500 MET-min/week) resulted in significant all-cause mortality benefits, with no evidence of further mortality benefits with increasing amounts of running [25]. Similarly, Ekelund and colleagues [26] in a systematic review and harmonized meta-analysis, recently examined the dose–response associations between accelerometer-assessed total physical activity, different intensities of physical activity, and sedentary time, and all-cause mortality in middle-aged and older adults. The authors revealed that any physical activity (regardless of intensity) and less time spent being sedentary were associated in a dose-dependent fashion with a significantly reduced risk for premature mortality [26]. Collectively, these findings support the importance of regular physical activity and/or exercise participation, reinforcing the straightforward health promotion motto to simply “***Move More, Sit Less***” [2].

From the outset of this editorial, it is important to acknowledge that health is not simply the absence of disease [4,27,28,29]. As such, it is also important to recognize the significant benefits of routine physical activity on wholistic wellness (e.g., spiritual, emotional, mental, and physical wellbeing) [4,27,28,29].

## 3. A Strengths-Based Approach and Effective Knowledge Mobilization

As discussed above, the health benefits of physical activity have been widely disseminated around the world. The varied national and international recommendations and guidelines for apparently healthy individuals and persons living with chronic medical conditions highlight the perils of too little activity and often have deficits-based statements contained therein or within supporting documents. For instance, many national and international physical activity guidelines provide threshold-based messaging suggesting that health benefits can only be accrued when a certain threshold for moderate-to-vigorous- (150 min/week) or vigorous- (75 min/week) intensity physical activity is achieved. This threshold- and expert opinion-based messaging is widely promoted despite strong evidence that significantly smaller volumes of physical activity (e.g., half or even less) can lead to marked and clinically relevant health benefits [4,20,23,30]. Moreover, a significant knowledge translation error can be introduced when threshold-based messaging is used, despite the evidence that demonstrates a curvilinear relationship with no clear threshold of health benefit [2]. Despite the strong evidence demonstrating the curvilinear relationship between physical activity and health outcomes, in recent years, statements such as “you need to do”, “you must”, or “you must engage in at least” have often been included in physical activity guidelines replacing more evidence-based statements that reflect the continuum of benefits by simply becoming more physically active [2,4].

Recently, a series of influential updated physical activity guidelines have been released from the United Kingdom (UK) [10] and the United States (US) [8]. Contained within these important evidence-informed and consensus-based guidelines and/related resources is the explicit acknowledgement that small changes in physical activity behavior can lead to significant health benefits (particularly in the least physically active/fit individuals). For instance, the UK Guidelines [10] state “Evidence now demonstrates that there is no minimum amount of physical activity required to achieve some health benefits.” These guidelines also state “For good physical and mental health, adults should aim to be physically active every day. Any activity is better than none, and more is better still.” Similarly, the US Guidelines supporting document states “The scientific evidence continues to build—physical activity is linked with even more positive health outcomes than we previously thought. And, even better, benefits can start accumulating with small amounts of, and immediately after doing, physical activity… Adults should move more and sit less throughout the day. Some physical activity is better than none. Adults who sit less and do any amount of moderate-to-vigorous physical activity gain some health benefits.” Throughout both of these landmark guidelines (and related documents) is the explicit statement that health benefits can be accrued at relatively small volumes of physical activity. As outlined previously, a strong body of literature supports this recommendation.

In these new guidelines and/or supplementary resources and knowledge translation tools, remain statements that may suggest that a certain threshold of physical activity (e.g., 150 min/week of moderate-vigorous physical activity) is required for health benefits. Having been involved extensively in guideline development and knowledge mobilization initiatives, we recognize the inherent complexities of creating effective and evidence-based recommendations and related knowledge translation resources for the general public. We respectfully would recommend a slight variation in the knowledge translation of these guidelines to remove statements that would imply that a certain threshold of benefit exists. For instance, despite acknowledging that any activity is better than none throughout the guideline documents, the UK guidelines [10] also include somewhat contradictory threshold-based statements such as “adults should accumulate at least 150 min (2 1/2 h) of moderate-intensity activity (such as brisk walking or cycling); or 75 min of vigorous-intensity activity (such as running)…” A related Infographic titled “Physical Activity for Adults and Older Adults” also includes contradictory statements such as “Some is good, more is better” and “Be active at least 150 min moderate intensity per week.” Similar statements are also provided for other age groups [10]. These conflicting statements also exist within the new US guidelines. For example, the US guideline supporting Infographics include statements such as “at least.” These statements, however, are generally made with other more temperate terms such as “should” or “aim” that reflect the research evidence. As we have previously discussed, the introduction of terms such as “at least” implies that a threshold of benefit exists, which does not appear to be the intention of the UK or US guidelines, which, throughout their related resources, demonstrate the importance of simply becoming more physically active. As we discussed recently [2], various researchers have discussed the potential perils of threshold-based messaging for facilitating health behavior change at the individual and population levels [4,20,31,32,33]. We do not feel that this was the intent of the UK or US guidelines and would like to celebrate these groups for taking a big step in promoting the importance of becoming more physically active for everyone. In the future, it would be ideal to remove terms such as “at least” or “a minimum of” to reflect the importance of simply engaging in physical activity or exercise and to help ensure the consistency in knowledge translation. Terms such as “at least” or “a minimum of” come from a deficits-based perspective and do not give full recognition of the significant capacity for positive change that exists within individuals or the benefits associated with very small changes in behavior.

When examining national and international physical activity guidelines, it is clear that most recommendations are by their nature externally focused and do not take into account the unique strengths and attributes of individuals. It can be argued that physical activity guidelines and the way that they are translated are therefore, by default, deficits-based, focusing on the problems in society and the risks associated with physical inactivity. Also, these guidelines often provide tips on how to help people avoid the health risks of being physically inactive. In the field of physical activity and health, it is also commonplace for deficits-based statements to be made that reflect the perils of engaging in too little physical activity and/or exercise. For instance, at keynote addresses and commentaries given throughout the world, it is not uncommon to hear statements such as:

- Children today will have a shorter lifespan than their parents.
- If you are inactive or sedentary, your chances of dying prematurely rise markedly.
- You must attain a certain level of physical activity to achieve health benefits.
- Our children and adults are receiving failing grades with respect to physical activity participation.

All of these statements are deficits-based in nature and, in certain cases, may not be supported by a strong body of evidence [34,35].

Various agencies have been working towards increasing the uptake of the physical guidelines; however, to date, there is relatively limited support for the efficacy of guidelines in creating large-scale population awareness [36,37,38] or health behavior change [2]. As such, it could be argued that current physical activity promotion approaches (often largely linked to generic physical activity guidelines) are not meeting the desires of contemporary society [27]. Berry et al. [38] recently argued that physical activity recommendations (based on strong epidemiological evidence) “largely fail to guide the choices that people currently make about” physical activity. Remarkably, there is even limited utilization of these guidelines by practitioners [31,32,39] and organizations with a clear invested interest in these guidelines [40]. Rutten and colleagues [11] also recently stated that the impact of national physical activity guidelines on “national-level policy-making might be considered modest at best.” Clearly, a better approach to the promotion of the health benefits of physical activity is required to support enhanced health and wellness at the individual and population levels. This is particularly salient, given the millions of dollars spent globally in the development of evidence-informed physical activity guidelines.

As we recently argued [2], the poor uptake of physical activity guidelines may (at least in part) be a result of an overreliance on health outcomes and physical activity dosages required to reduce risk (i.e., deficits-based messaging) rather than effectively addressing the key determinants of healthy lifestyle behaviors. This supports the recent movement to engage in knowledge mobilization activities (e.g., the creation of knowledge translation resources) that highlight evidence-based means of promoting physical activity/exercise enjoyment and adherence [2,41,42,43,44]. For instance, Rhodes and colleagues (and others) have demonstrated strong support that affective judgment constructs (such as affective attitude, enjoyment, intrinsic motivation) are key predictors of physical activity behavior [42,43,44]. Exercise enjoyment particularly appears to be a key determinant of behavioral change related to healthy lifestyle behaviors (such as physical activity) [41]. A growing body of research has supported the efficacy of promoting the positive affective benefits of physical activity (such as “feeling good”) for eliciting behavioral change. Accordingly, it has been argued that current physical activity recommendations should have a greater focus on affective constructs [2]. We have also advocated a transdisciplinary, individualized, and person-centered approach to support healthy lifestyle behaviors [2,4].

Increasingly, health agencies and practitioners have recommended moving away from deficits-based recommendations and/or approaches to health and wellness promotion. Strengths-based approaches to health and wellness have gradually been advocated [45,46,47]. Strengths- (or assets-) based approaches are internally focused, building upon the aspirations, innate strengths, and opportunities/resources of individuals, families, and communities serving as the foundation to build upon to improve health and wellness [47,48,49,50]. As outlined recently by Zhang and colleagues [50], a strengths-based approach includes the client’s resources and preferred future rather than focusing on the past histories or health problems (deficits). Therefore, this approach is not focused on the cause of the problem, but rather on the past successes and what a client can and/or will do to enhance his/her own health and wellbeing in the future [47,50].

At its core, strengths-based approaches are wholistic and transdisciplinary [47], involving the active engagement of the individual, the individual’s network (e.g., family and community), and others (such as healthcare professionals and providers, qualified exercise professionals) [47]. A respectful co-creation approach is taken where practitioners work in collaboration with and under the direction of the client [50]. This approach allows for the support of individuals, families, and communities in making independent and informed decisions about health and wellness. This person-centered method has the potential to facilitate self-empowerment and self-determination related to health and wellness, supporting the positive effects of being physically active, while minimizing the negative feelings often associated with physical activity promotion (e.g., shame, inadequacy, stigma).

There are a variety of strengths-based approaches (e.g., solution-focused therapy, strengths-based case management, asset-building model of community management, individual placement and support model of supported employment) and tools available [47,51,52]. As reviewed by Pattoni [51] strengths-based practice is a collaborative process involving individuals and others supporting them. Together, they work towards identifying a desired outcome that builds upon the inherent strengths and assets of the client. In this collaborative model, individuals are not simply consumers of health services but, rather, co-producers of the services that support their desired future outcomes [51], drawing upon their past successes in addressing their own issues and goals [50]. Rapp and colleagues [52] outlined six hallmark standards for effective strengths-based approaches. We have adapted these categories to highlight how this approach can be used in physical activity and health and wellness promotion settings.

1. **Goal Orientation:** A strengths-based approach is foremost goal-oriented and person-centered. In this phase, clients establish the goals for their life. For instance, clients could establish the goals that they would like to achieve related to physical activity, exercise, health, and wellness in their future. A practitioner can be an active participant in the discussion of these goals, but should allow clients to fully articulate their desires and aspirations [52] related to health and wellness and life in general.
2. **Strength Assessment:** The clients are supported to recognize the inherent strengths and resources at their disposal that can be used to offset any difficulty or condition. This often relates to current strengths; however, the past may be mined for previous strengths (assets, talents, resources) that may have been lost or forgotten. It is essential to focus on the strengths of each person and not on the problems, deficits, or disease condition [52]. For example, clients that aspire to become healthier can recognize the activities that they enjoy, what works for them, and the opportunities for doing these activities with their family and within their community.
3. **Resources from the Environment:** This refers to how a person’s environment can be rich in resources that allow the client to achieve his/her aspirations. This includes individuals, groups, associations, and institutions that may provide resources and/or support for the client. A practitioner can serve as the conduit (linkage) to these resources [52]. For instance, a client desiring to become more physically active may contact a qualified exercise professional to determine the resources available within the community related to physical activity. The exercise professional can assist the client in identifying the opportunities and resources available for becoming more physically active [31].
4. **Explicit Methods Are Used for Identifying Client and Environmental Strengths for Goal Attainment:** There are a variety of strengths-based approaches to meet the goals and aspirations of the client. There will be subtle differences in how each of the strengths-based techniques will be applied [52]. For instance, solution-focused therapy has been increasingly used within clinical settings [50], wherein clients will set goals and then identify relevant strengths (such as what works now, what may work in the future) [52]. In strengths-based case management approaches, clients will go through a tailored “strengths-assessment” that assists the client in establishing goals, generating resource options and opportunities, setting short-term goals and tasks, and directing roles and responsibilities [52]. In cardiac rehabilitation and exercise settings, it is not uncommon for practitioners to make use of different strengths-based approaches to support clients in enhancing their health and wellbeing [16].
5. **Relationship is Hope-Inducing:** Strengths-based approaches are designed to enhance the hopefulness of the client. Hope can be realized by finding strengths and through empowering relationships with others, communities, and/or culture. This process allows clients to increase their perceptions regarding their abilities, enhance clients’ options and perceptions of these options, and increase the confidence and opportunities of clients to choose and act on these choices [52]. In physical activity promotion, identifying strengths related to physical activity participation and connecting with other people, communities, and culture can build hope towards enhancing health and wellbeing [48].
6. **Meaningful Choice:** Central to a strengths-based approach is the belief that clients are the experts in their own lives. The practitioner’s role is to enhance and explain choices, encouraging clients to make their own informed decisions and choices [51,52]. Through a strengths-based approach in physical activity promotion, we can support self-empowerment and self-determination wherein clients have control over their health and wellbeing. By being active participants in their own health and wellbeing, there are also greater learning opportunities for clients, such as facilitating a greater understanding of the importance of routine physical activity participation for health and wellness.

**Figure 1:**
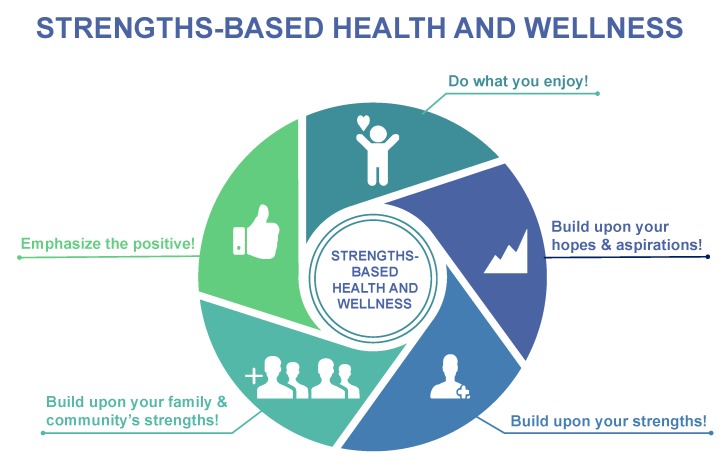
Strengths-based health and wellness promotion through physical activity.

A strengths-based approach supports various recent international and regional health authorities mandates for person-centered care. For instance, the Canadian Institutes of Health Research have recently highlighted the importance of person-centered research wherein patients, researchers, healthcare practitioners, administrators, and policy-makers work collaboratively together to determine and focus upon patient identified priorities to improve patient outcomes [53]. The Department of Health and Social Care in the United Kingdom [47], in a recent report regarding the application of strengths-based approaches in social care settings, articulated the capacity for strengths-based approaches to be used in all professions with appropriate attention and care. In the strengths-based approach, practitioners work in a collaborative way to get a better understanding of the individual’s abilities, aspirations, and current circumstances [47]. Practitioners working with clients will work together to identify the best steps for health change, making use of the client’s inherent strengths, aspirations, and resources. The practitioner also helps ensure that the appropriate tools are available to support the client in his/her health and wellness journey [47]. Figure 1 outlines how a strengths-based approach to health and wellness promotion through physical activity can be taken that builds upon the hopes, aspirations, and strengths of individuals and their families and communities. In Table 1, we outline how deficits-based messaging and/beliefs can be reframed to take a strengths-based approach recognizing the inherent strengths of individuals and their families and communities.

The application of strengths-based approaches has increasingly been used in diverse environments (such as the workplace and clinical settings [45,50,52,54,55]), communities, and populations (e.g., children, Indigenous communities) [48,49,56,57,58,59]. For instance, Zhang et al. [50] in a recent systematic review and meta-analysis of randomized controlled trials, revealed that strengths-based, solution-focused brief therapy in medical settings had a significant effect on health-related psychosocial outcomes (e.g., depression, psychosocial adjustment to illness), with positive indicators for health-related behavioral outcomes (e.g., physical activity, nutrition score). Another systematic review by Tse and colleagues [60] examined the effectiveness of strengths-based interventions for people with serious mental illness. Although there was relatively limited literature, the authors found emerging evidence supporting strengths-based approaches in clinical settings, including improved outcomes such as hospitalization rates, employment/educational attainment, and intrapersonal outcomes (e.g., self-efficacy and sense of hope).

Traditionally, physical activity recommendations and exercise prescriptions have taken deficits-based approaches including identifying an issue and creating recommendations/prescriptions to address/fix the issue. We advocate the widespread usage of strengths-based methodologies in cardiac and other clinical exercise rehabilitation settings. This belief system has affected directly our own practice. For instance, we have demonstrated that an individualized and patient-centered approach was a safe and efficacious way to improve the health and wellbeing of persons living with major mental illness [61]. Recently, we have adopted a strengths-based approach that serves to enhance the self-empowerment and self-determination of persons living with chronic medical conditions. Similarly, in cardiac rehabilitation settings, the avoidance of generic physical activity guidelines is advocated, with a more individualized and person-centered approach to health and wellness being taken [16]. As outlined by Kabboul and colleagues [62], the core components of cardiac rehabilitation include nutritional counseling, risk factor modification, psychosocial management, patient education, and individualized exercise training. Each cardiac rehabilitation program is distinct, with many making extensive usage of strengths-based approaches building upon the aspirations and resources of the clients. We advocate that international cardiac rehabilitation agencies (and other exercise rehabilitation programming for other medical conditions) support the greater inclusion of strengths-based and person-centered approaches to enhancing the health and wellness of patients.

Strengths-based approaches to health and wellness have increasingly been used within Indigenous communities throughout the world [48,49,57,58,63]. Indigenous peoples have acknowledged the need to consider health and wellness from a more wholistic perspective (including spiritual, emotional, mental, and physical wellbeing) reflecting a balance of human relationships with the natural and spiritual world, including connections amongst the land, individuals, family, community, and cultural and spiritual practices [63]. For instance, a recent report by Interior Health (British Columbia, Canada) recommended moving towards a wellness model that integrates traditional Indigenous approaches into best practices [64]. In this model, Indigenous culture, spirituality, and connection to family, community, and land are important foundations of healing and wellness [65]. Health and wellness approaches should not solely focus on the individual, but also affect the family and the community as a whole [64]. Wholistic lifelong learning models have also demonstrated that learning from and about language, culture, and tradition is essential for the wellbeing of Indigenous peoples [66]. We have also recently demonstrated that cultural identity was an important determinant of health among Indigenous Canadians [67].

Indigenous peoples often consider healthcare models based on Western (colonial) belief systems as being associated with the potential for harm, rather than promoting healing or wellness [64]. Growing evidence reveals that the experiences of many Indigenous peoples with mainstream healthcare systems are often negative due to cultural differences and/or insensitivity [68,69]. The failure to address these cultural differences or insensitivity has often been associated with lower rates of compliance with health advice, a relative reluctance to use healthcare facilities, feelings of fear, a lack of respect, and estrangement within Indigenous peoples [68,69]. Ensuring cultural competencies and the creation of culturally safe places has been widely promoted to help enhance health and wellness within Indigenous peoples [68,69].

For more than 20 years, we have worked in Indigenous communities throughout Canada. This includes partnering with more than 350 Indigenous communities (>10,000 participants) on healthy lifestyle and wellness programs that are led by Indigenous community leaders in collaboration with Indigenous trainees, youth, and scholars [48,70]. Through this work, we have demonstrated the remarkable efficacy and effectiveness of strengths-based approaches to wholistic health and wellness that build upon the expertise, experiences, knowledges, aspirations, and skills of Indigenous peoples [48,70]. This Indigenous-led, community-based, participatory research revealed clearly the importance of focusing on the inherent strengths and aspirations of the family and community, rather than the problems (or deficits). In this strengths-based approach, health and wellness are built from within, wherein everyone is a teacher and a learner [71].

In our experience, a strengths-based approach to health and wellness resonates strongly with Indigenous Elders, leaders, and community members, consistent with Indigenous ways of understanding and doing. It was also clear that a one-size-fits-all approach does not work within Indigenous communities. For instance, generic physical activity guidelines were deemed as not being culturally appropriate or safe [48,70]. Also, by using generic (one-size fits all) guidelines, we are not able to focus on the aspirations and the inherent strengths of individuals and their families and communities [1]. We also recognized that traditional Indigenous wholistic healing approaches (such as using collective strengths, gaining strength via spirituality, cultivating cultural identity) that affect the wellness of the entire community should be explored further [67]. We also identified a series of knowledge translation and healthcare gaps that exist within Indigenous communities. We recognized the relative lack of understanding by healthcare professionals regarding the traditional health and wellness practices of Indigenous peoples and the lack of integration of this knowledge within Western medicine models [69]. Addressing this knowledge gap requires a culturally responsive approach that accommodates the values of Indigenous peoples, their patterns of communication and behavior, their histories of living and learning, and the sources of knowledge that they give priority to [72]. By creating a culturally responsive, accessible, and safe program, we are able to enhance the engagement of Indigenous peoples and bridge cultural and mainstream scientific knowledge [72].

In our work, we incorporate co-creation design [48,70] to provide insight into the strengths, aspirations, and resources of individuals and their families and communities. Using co-creation design has helped ensure that we are well positioned to capitalize on a strengths-based approach for sharing wholistic perspectives of health and wellness unique to the historical, traditional, and cultural perspectives of Indigenous communities. This includes the extensive usage of Indigenous-led sharing circles as the key means of the exchange of wisdom, cultures, experiences, and knowledge systems unique to Indigenous peoples. Sharing circles honor Indigenous oral history and storytelling traditions, allowing the participants to engage in narrative as a way to draw out their thoughts and ideas rather than imposing motivation and commitment [73,74]. For instance, Elders will often tell stories as a form of oral teaching to share culture, traditions, and history. Ideas are being actively shared, and participants have the opportunity to find new meaning with each story. Teaching and learning occurs naturally in both directions. These cultural sharing circles provide knowledge regarding Indigenous understandings and the complex social, cultural, historical, and economic factors that shape health and wellness. Sharing circles also ensure that each person has the opportunity to share and that no one is passed by [48].

Consistent with health and wellness practices advocated through the Truth and Reconciliation Commission [75], strengths-based research is a culturally relevant strategy, supporting experiential learning and allowing an individual to gain resiliency through self-empowerment and self-determination [51]. Because the intergenerational effects of Indian Residential Schools within Canada have led to maladaptive coping mechanisms affecting physical, mental, emotional, and spiritual wellness [76], a strengths-based style of research is an appropriate strategy for empowering individuals to take ownership of their health using culturally safe and relevant methods. We recognize that Indigenous community leaders and Elders provide a wealth of shared and lived experiences related to Indigenous self-government, language revitalization, community engagement, traditional Indigenous health and wellness practices, connectedness to land, culturally relevant and safe messaging, spiritual health and wellness, and Indigenous-led and community-based approaches to health and wellness. Ultimately, our strengths-based approaches are intended to enhance the empowerment and self-determination of Indigenous peoples, providing greater ownership over health and wellness within their community and combining both traditional and Western health practices [65]. By taking this approach, we have observed the significant capacity of Indigenous peoples to exert control over their own health and wellness [48,70].

## 4. Conclusions

Through our special series on Cardiac Rehabilitation [77], we highlighted the overwhelming evidence supporting the importance of routine physical activity and/or exercise participation in the primary and secondary prevention of cardiovascular disease and many other chronic medical conditions. The health benefits of routine physical activity have been widely disseminated; however, this messaging is often associated with deficits-based recommendations that highlight the risks of not engaging in sufficient amounts of physical activity. This deficits-based information often includes generic threshold-based recommendations suggesting that health benefits can only be accrued at specific volumes or intensities of physical activity. In this Editorial, we advocate for moving away from externally and problem-focused (deficits-based) messaging and approaches that have often defined physical activity, exercise medicine, exercise science, and cardiac rehabilitation fields. We advocate a strengths-based approach to health and wellness promotion that focuses on the innate strengths of individuals, families, and communities. This is a person-centered approach, supporting self-empowerment and self-determination related to health and wellness. An increasing body of research from diverse environments (such as clinical settings, workplaces, and communities) has shown the efficacy and effectiveness of assets-based approaches to health and wellness. In particular, work within Indigenous communities has shown the ability of the strengths of individuals, families, and communities to cause real change in health and wellness at the individual, family, and community levels. This includes community-based and Indigenous-led research that demonstrates the remarkable capacity for individuals to be the key advocates for health and wellness change. We feel strongly that the lessons learned from Indigenous peoples focusing on the inherent strengths of individuals, families, and their communities apply directly to the global desire to improve the health and wellbeing of contemporary society.

## 5. Key Take-Home Message

By taking a strengths-based approach, we can build hope, facilitating trust in one’s own expertise and decisions, promoting the positive aspects of routine physical activity and exercise participation. Ultimately, this strengths-based approach (building upon the aspirations and the inherent strengths of individuals and their families and communities) will provide a greater opportunity to enhance health and wellbeing for everyone.

## Figures and Tables

**Table 1:** Reframing weaknesses as strengths in physical activity promotion.

Weaknesses (Deficits-based)	Strengths
Instead of These Weakness Statements…	…Try These Strength Statements
***Population-based Messaging***	
Adults should engage in at least 150 min of moderate to vigorous physical activity on a weekly basis.	We are excited about the potential for small changes in physical activity to lead to marked health and wellness benefits, particularly in inactive individuals. Simply by moving more, we can improve the health and wellbeing of society.
Our children today will die at a younger age than their parents.	We have a great opportunity to address challenges such as obesity to enhance the health and wellbeing of our children through physical activity and other healthy lifestyle behaviors.
Children and adults have received a failing grade with respect to physical activity.	Many children and adults are active and reaping the benefits of routine physical activity. Building on the inherent strengths and resources available to children and adults, we can work together to meet the goal of being healthier and feeling better.
If you are inactive or sedentary your chances of dying prematurely rise markedly.	By moving more and sitting less, we can achieve the goal of a healthier society.
***Person-centered Messaging***	
Like the majority of Canadians, I am physically inactive and therefore do not get all of the benefits associated with physical activity.	I look forward to building on my strengths, to become more active so that I can achieve the health and wellness benefits associated with physical activity.
I live with a chronic medical condition and am fearful of engaging in exercise.	I am happy to hear that physical activity is of benefit and quite safe for virtually everyone, including people living with chronic medical conditions.
I have been told that in order to achieve health benefits, I must engage in at least 150 min of moderate-to-vigorous physical activity on a weekly basis.	I am excited to know that small changes in physical activity levels can lead to significant health benefits. By making small changes in my activities, I can meet my goal to be happier and healthier.
I do so little physical activity daily that it is hard to imagine that I could meet international recommendations.	I realize that I do not need to engage in large amounts of physical activity to take control over my health and wellbeing.
My lifestyle habits and patterns have made me weak and susceptible to dying early and developing several chronic medical conditions.	I will build upon my strengths and the support of my family to be healthier. If I make small changes, one at a time, I will surprise myself by how much I can achieve.
I find many physical activities not enjoyable.	I will focus on physical activities that are enjoyable, so I can experience the multiple benefits of being physical active.
I do not enjoy exercising on my own.	My family and friends are excited to help me on my journey to become more physically active.
In the past, I have doubted myself about my ability to become more physically active on a routine basis.	I have a strong conviction to be more active and healthier. I am excited about my potential to be more active and healthier for years to come.
I do not feel confident about my ability to be physically active.	Looking at my inherent strengths and the support of my family and community, I feel empowered to become more physically active.
I feel helpless and do not know what to do.	Building on my strengths and available support systems, I can have greater control over my health and wellbeing. I have a strong sense of hope and optimism for my future.
I do not have enough time.	I can take the limited time that I have available to make small changes in my activity patterns.
When working with exercise professionals in the past, I have had little control over my activity programming.	By working together with exercise professionals, we can discuss my aspirations so that I am empowered to be more active and healthier.
I do not have a good understanding of the best way of becoming more physically active.	I look forward to working with experts and other members from my family or community on exploring the best ways to become more active and healthier.
I do not know of all of the opportunities available to me in my community.	Working together with my family, others from my community, and/or practitioners, I can gain a greater understanding of the physical activity resources that are available to me within my own community.
I cannot afford the costs associated with being physically active.	I am excited to engage in free activities that can be done within my own community with my family and friends.
I am fearful of the challenges associated with exercising with others.	I am hopeful of the strengthened relationships that I can develop through physical activity with my family and community.
